# The dual role of metabolic reprogramming in macrophage polarization in rheumatoid arthritis and coronary heart disease and the intervention strategy of traditional Chinese medicine

**DOI:** 10.3389/fcvm.2026.1874530

**Published:** 2026-06-30

**Authors:** Haichun Zheng, Xiuyue Li, Jing Ru, Zhuoran Meng, Weibing Liu, Tao Jia, Shan Zhang, Qianwen Deng, Wenyi Huang, Renjie Dong, Qianrong Li

**Affiliations:** 1 School of Basic Medical Sciences, Yunnan University of Traditional Chinese Medicine, Kunming, Yunnan, China; 2 The First People's Hospital of Yunnan Province, Kunming, China; 3 Department of Orthopedics, First Clinical Medical College of Yunnan University of Traditional Chinese Medicine, Kunming, Yunnan, China; 4 Yunnan Key Laboratory of Integrated Traditional Chinese and Western Medicine for Chronic Disease in Prevention and Treatment, School of Basic Medicine, Yunnan University of Chinese Medicine, Kunming, Yunnan, China

**Keywords:** atherosclerosis, coronary heart disease, macrophage polarization, metabolic reprogramming, rheumatoid arthritis, traditional Chinese medicine

## Abstract

Macrophage polarization has become increasingly acknowledged as a process that is governed by metabolism which will have significant impacts on long-term inflammatory and cardiovascular diseases. Through the atherosclerotic component of coronary pathology, both rheumatoid arthritis and coronary heart disease result in macrophages undergoing dramatic metabolic changes that ultimately determine how they functionally behave in terms of their inflammatory characteristics. While pro-inflammatory M1 macrophages exhibit a preference for aerobic glycolysis, anti-inflammatory/tissue repair M2 macrophages are more heavily reliant upon oxidative phosphorylation/fatty acid oxidation. The balance between these two types of metabolism is typically disrupted towards the glycolysis dominant type in both rheumatoid arthritis and atherosclerosis; thus contributing to increased inflammation, sustained inflammatory amplification, and exacerbated tissue damage. Metabolic regulators involved in modulating the glycolytic metabolism of macrophages include Pim2 kinase (in rheumatoid arthritis), phosphoglycerate kinase 1, pyruvate dehydrogenase E1 alpha 1 (in rheumatoid arthritis), and granulocyte-macrophage colony stimulating factor (in rheumatoid arthritis). Furthermore, in coronary heart disease, activation of the PI3K/AKT pathway increases the programming of glycolytic metabolism in macrophages and increases plaque inflammation/lesion instability while the oxidative metabolism associated with M2 macrophages results in resolution/plaque stability. Given this context, there is an increasing amount of evidence demonstrating that certain compounds found within traditional Chinese medicines, including berberine, ginsenoside Rb1 and formulae targeted at the HIF-1α/PDK1 axis can be used to re-balance macrophage metabolism to induce a shift away from inflammatory dominance. This review focuses on the dual roles of macrophage metabolic re-programming in rheumatoid arthritis and coronary heart disease, compare common/disease specific signaling components such as HIF-1α, AMPK, and mTORC1 and evaluate the therapeutic potential of traditional Chinese medicine for restoring immunometabolic homeostasis.

## Introduction

Traditionally, studies have focused on rheumatoid arthritis (RA) and coronary heart disease (CHD) as separate clinical areas of study. Rheumatoid arthritis has dealt primarily with chronic autoimmune synovitis leading to progressive damage of joints, while coronary heart disease has addressed the process of atherosclerosis and the occurrence of ischemic cardiac events (1, 2). However, there is increasing evidence that RA and CHD are linked through common biological pathways involving shared processes of inflammation and metabolism (3). Therefore, patients with RA are at significantly increased risk of experiencing a cardiovascular event; however, an individual's overall risk may not be entirely explainable based solely upon traditional risk factors (4). Rather, chronic systemic inflammation and the activation of immune cells, along with impaired function of the endothelium, oxidative stress, and alterations in how lipids are processed can all contribute to a “pathogenic bridge” connecting the disease process occurring within the synovium to the development of atherosclerosis (5). In this context, macrophages play a particularly significant role. Macrophages represent a large population of effectors involved in both the inflammatory process of the affected synovium and in the development of atherosclerotic lesions (6). Additionally, due to their high degree of metabolic plasticity, macrophage phenotypes are highly responsive to the biochemical environment of the tissue microenvironment. Due to these characteristics, studying macrophage biology will provide unique insight into the relationship between RA and CHD.

Macrophage polarization has dramatically changed our present view of chronic inflammatory diseases (7). Macrophages are no longer viewed as static phagocytes that function under a single role, rather they can dynamically respond to various stimuli including cytokines, metabolites, O2 tension, nutrient availability, and cellular derived factors to adopt many different forms of activation. Although the simplistic M1/ M2 model is still helpful for providing an overview of macrophage polarization (classically activated M1 like macrophages are generally involved in the promotion of inflammation and damage to tissues by their ability to generate pro-inflammatory mediators and antimicrobial defenses vs. alternatively activated M2 like macrophages are generally involved in anti-inflammatory processes such as clearance of dead cells through efferocytosis, and repair of damaged tissues through matrix remodeling) it is important to recognize that the biological relevance of this model exists beyond just differences in cytokine production and receptor expression (8). This is because the type of metabolism used by a specific macrophage population is tightly linked to the type of macrophage population (9). For example, pro-inflammatory macrophages use an aerobic glycolytic pathway, which is similar to what occurs during the Warburg effect, and is characterized by the production of ATP at high rates, and the production of biosynthetic intermediates necessary for inflammatory mediator synthesis (10). On the other hand, reparative macrophages produce ATP using oxidative phosphorylation in mitochondria and fatty acid oxidation, both of which result in high levels of energy efficiency, redox homeostasis, and promote resolution of inflammation (11).

In rheumatoid arthritis (RA), the role of synovial macrophages in creating and maintaining the inflammatory environment, recruiting other immune cells into the joint space and enhancing the cytokine cascade contributing to the generation of pannus, cartilage degeneration and bone destruction are significant. The RA synovium represents a hostile metabolic environment in terms of hypoxia, high levels of pro-inflammatory cytokines and alterations in nutrient utilization (12). As a result of their metabolic environment, macrophages undergo a transition from oxidative phosphorylation to glycolytic metabolism and become polarized towards an inflammatory phenotype (13). Consequently, they continue to support chronic inflammation within the synovium and long-term joint damage. Studies using mechanistic approaches suggest that macrophages' metabolic changes are not passive events; instead they are tightly regulated by signaling pathways and metabolic enzymes (14). For example, it has recently been demonstrated that Pim2 kinase can promote glycolysis and induce an M1 like-polarization response in macrophages via phosphorylation of important metabolic enzymes (e.g., phosphoglycerate kinase 1) and pyruvate dehydrogenase E1-alpha-1 (15). Moreover, the granulocyte-macrophage colony stimulating factor has induced macrophages that express high levels of metabolic enzymes and thus support prolonged inflammation and RA activity (16). Collectively, the above data have allowed researchers to redefine RA as not only a cytokine-driven autoimmune disease but rather an immunometabolic disease in which macrophage preference for different fuels supports the disease's pathologic consequences.

A similar metabolic paradigm is also becoming apparent in atherosclerosis, which is the underlying pathologic basis for most coronary artery diseases. The primary characteristics of an atherosclerotic plaque include not only lipid accumulation, but also complex interactions among dysfunctional endothelium, retained lipoproteins, oxidative stress, dead cells, and chronic inflammation (17). The involvement of macrophages has been shown to be important at all stages of plaque development, including early monocyte recruitment and the subsequent transition into foam cells, necrosis within the core of the plaque (necrotic core), the failure of efferocytosis, disruption of the fibrous cap of the plaque leading to increased thrombogenic potential (18). Similar to what was found in rheumatoid arthritis, there appears to be strong correlation between macrophage phenotypes present in atherosclerotic plaques and their metabolic states. Glycolysis promotes M1-like macrophage activation, increases production of cytokines involved in the inflammatory process and thus can promote both progression and instability of an atherosclerotic plaque (19). On the other hand, oxidative phosphorylation and fatty acid oxidation may have more direct associations with M2-like functions of macrophages that would enhance processes of efferocytosis, resolution of inflammation, and stabilization of an atherosclerotic plaque (20). The PI3K/AKT signaling pathway influences these macrophage metabolic orientations through its role in stimulating glycolysis and inflammatory responses (21). In addition, this pathway links intracellular sensing mechanisms for nutrients to behaviors exhibited by the atherosclerotic lesion. Therefore, the macrophage present within an atherosclerotic plaque does not simply respond to tissue injury; rather it is metabolically programed to determine whether the atherosclerotic lesion will remain stable or become unstable and ultimately lead to a clinically relevant coronary event.

The vast majority of research on TCM has been conducted in a manner which utilizes the specific terminology associated with TCM. However, there is growing evidence from pharmacologic studies that demonstrate that many of the active monomers and formulations used within TCM are capable of affecting the biochemical pathways responsible for influencing the polarization of macrophages (22). For example, berberine activates AMPK and therefore promotes a metabolic profile that favors less inflammation by macrophages (23). Similarly, ginsenosides (such as ginsenoside Rb1) have shown potential as mediators of inflammatory signal transduction, oxidative stress, and macrophage phenotypes (24). Quercetin acid, when used to treat rheumatoid arthritis via nano-delivery systems based on Gentiana macrophylla, have targeted the ERK/HIF-1α/GLUT1 pathway; suppressed glycolytic activity; promoted the transition of M1 macrophages to M2, etc (25). Collectively these data suggest that traditional Chinese medicine does not act solely in a symptomatic or adjunctive fashion, but potentially possesses mechanistic relevance as a regulator of macrophage immunometabolism.

The relevance to translation of this approach is significant. Individuals with RA who also have CHD are an especially difficult clinical population in that they exhibit overlapping pathophysiology (inflammation, endothelial dysfunction, abnormal lipid metabolism, immune suppression) that will likely worsen outcomes for each of their conditions. In contrast to many Western medical therapeutic approaches, traditional Chinese medicine has generally focused on treating RA and CHD separately from one another as opposed to utilizing a unifying biological strategy to treat them simultaneously. Thus, the integration of traditional Chinese medicine into modern day immunometabolism may be best achieved through a literature review that combines these two fields. Therefore, such a literature review could potentially offer more than just a description of these fields. It could provide clarity regarding the molecular mechanisms by which inflammation resulting from RA accelerates CHD, identify metabolic reprogramming of macrophages as a common mechanism underlying both diseases, and determine if treatments based on traditional Chinese medicine may be able to restore a more balanced state between inflammatory macrophages driven by glycolytic pathways and oxidative repair-oriented macrophages.

## Immunometabolic basis of macrophage polarization

### Beyond the M1/M2 binary: a continuum of macrophage phenotypes

Before discussing the metabolic features that distinguish pro-inflammatory and reparative macrophages, it is important to acknowledge that the classical M1/M2 dichotomy, while still heuristically useful, represents an oversimplification of the remarkable phenotypic diversity observed *in vivo*. Macrophages exhibit a large repertoire of activation states, which arise from multiple combinations of macrophage-activating factors rather than falling neatly into two discrete categories (26). As Murray et al. have argued, the field requires a standardized nomenclature and experimental guidelines that move beyond the simplistic M1/M2 model, while still recognizing that the two poles provide a useful operational framework for comparing different activation states (27). Different stimuli—ranging from cytokines and microbial products to metabolic and viral signals—can drive macrophages into distinct functional states. For instance, influenza A virus (H1N1) infection of human monocytic cells induces specific metabolic alterations, including reduced glucose utilization, demonstrating that viral stimuli produce an activation state distinct from classical LPS/IFN-γ-driven M1 polarization (28). Similarly, glucocorticoids can induce a unique gene expression program in mature macrophages via upregulation of TGF-β receptor II, leading to an activation state that shares some features with M2-like cells but is not identical to IL-4-induced M2 polarization (29). Thus, macrophages *in vivo* exist along a continuous spectrum of activation states, with the M1 and M2 poles representing only the extremes of a much broader phenotypic landscape.

Importantly, the recognition of this continuum does not invalidate the observation that the two extreme poles of activation—classically activated (M1-like) and alternatively activated (M2-like)—are associated with fundamentally different metabolic programs. The M1-like pole is consistently characterized by a shift towards aerobic glycolysis, while the M2-like pole relies more heavily on oxidative phosphorylation and fatty acid oxidation. However, macrophages that fall between these extremes may exhibit intermediate metabolic profiles: they might show partial upregulation of glycolytic enzymes without full commitment to the Warburg effect, or they may retain some oxidative capacity while still producing inflammatory cytokines. Therefore, in the following sections we use the terms “M1-like” and “M2-like” as reference poles to describe the dominant metabolic orientation of macrophage populations in RA and CHD, while fully acknowledging that the precise metabolic phenotype of any given macrophage population depends on the specific combination of local stimuli.

### Glycolysis and the warburg effect in pro-inflammatory macrophage activation

A hallmark of the proinflammatory activation of macrophages is the preference for using aerobic glycolysis, which has been referred to as a “Warburg-type” (aerobic) shift in metabolism (30). However, the precise directionality of the relationship between glycolytic metabolism and M1 polarization remains an area of active investigation. While it is well established that pro-inflammatory macrophages predominantly utilize glycolysis, whether metabolic reprogramming per se drives the acquisition of an inflammatory phenotype or whether polarization signals dictate metabolic changes is still debated (31). In this condition, there is an increase in both glucose utilization and the rate of conversion through glycolysis, regardless of whether or not enough oxygen exists to sustain mitochondrial oxidative phosphorylation (32). Although this type of glycolysis would appear to be inefficient from a purely biochemical standpoint, it serves a biological advantage for activated macrophages during their inflammatory response (33). Glycolysis not only allows activated macrophages to rapidly generate ATP but also to continuously produce biosynthetic intermediates needed for *de novo* nucleotide synthesis, lipid remodeling, redox buffering and cytokine production (34). Therefore, the high rate of glycolytic flux is likely critical for activated macrophages to rapidly respond to their energetic and anabolic needs of inflammatory activation in hostile tissue environments (35). The inflamed synovium found in rheumatoid arthritis is characterized by hypoxia, competitive inhibition for nutrients and continuous cytokine signaling; all conditions that promote a glycolytic phenotype (36). Similarly, in atherosclerosis, macrophages are shifted towards a glycolytic phenotype due to the presence of inflammatory cytokines and oxLDL in addition to local environmental stresses (37). The glycolytic bias of the metabolic pathway in M1 like macrophages is highly related to their functional characteristics (31). The enhanced glycolytic rate is associated with an increased rate of production of proinflammatory mediators and sustained NLRP3 inflammasome-mediated signaling (38). Additionally, metabolites derived from glycolysis can feed into other metabolic pathways and modulate transcriptional and post-transcriptional regulatory programs that contribute to maintaining the expression of genes involved in inflammation (39). The glycolytic state has been proposed to function not merely as an energy-generating pathway but as a signal transduction architecture that stabilizes pro-inflammatory identity. This interpretation is supported by studies showing that pharmacological inhibition of glycolysis (e.g., using 2-deoxyglucose) suppresses the expression of pro-inflammatory genes in LPS-stimulated macrophages, suggesting that an intact glycolytic flux is necessary—but not necessarily sufficient—for full M1 polarization (40). Therefore, the Warburg-like phenotype in macrophages represents a strategic metabolic adaptation that enhances inflammation and contributes to the development of chronic disease states when resolution mechanisms fail.

### Oxidative phosphorylation and fatty acid oxidation in anti-inflammatory and reparative macrophage states

Unlike the glycolytic-based inflammatory response of macrophages; the non-inflamatory, reparative responses of macrophages have a greater reliance upon mitochondrial oxidative phosphorylation and fatty acid oxidation for their energy needs (41). These pathways provide a basis for sustained energy production, efficient ATP (adenosine triphosphate) formation, redox equilibrium, and longer term maintenance of homeostasis functions such as efferocytosis, matrix remodeling, angiogenic modulation, and repair of damaged tissues. Oxidative phosphorylation provides macrophages with energy generated by the process of mitochondrial respiration (42). The comparative metabolic features and related TCM interventions are summarized in Tables 1, 2. Although oxidative phosphorylation produces energy at a rate lower than that produced by glycolysis, it is significantly more efficient in its use of substrates. The presence of both acetyl-CoA and reducing equivalents necessary to fuel the tricarboxylic acid cycle and electron transport chain are provided by fatty acid oxidation (43). Macrophages which can utilize this oxidative pathway will be characterized by M2-like behavior, i.e., they will produce anti-inflammatory mediators, promote resolution, and support the regeneration of damaged tissues (44). However, the notion that fatty acid oxidation (FAO) is uniformly reparative has been challenged by recent studies. Notably, in the infarcted heart following myocardial infarction (MI), cardiac macrophages residing in the infarct zone paradoxically upregulate the *de novo* fatty acid synthesis pathway via ACLY and FASN, rather than relying predominantly on FAO. Myeloid-specific deletion of Acly or Fasn improved post-MI cardiac function and reduced fibrosis, demonstrating that in this specific pathological context, endogenous fatty acid synthesis in macrophages is pathogenic and drives pro-fibrotic outcomes. Moreover, ACLY was shown to acetylate the promoter region of Krt17, driving the production of pro-fibrotic cytokines including IL-33, which in turn expands a pathogenic fibroblast subset (45). These observations add important nuance, indicating that the role of macrophage lipid metabolism in tissue repair is context-dependent and cannot be reduced to a simple FAO-good paradigm. The utilization of this oxidative pathway has been linked to increased stability of plaques in atherosclerotic disease, enhanced efferocytosis of apoptotic cells, and decreased amplification of inflammatory signals. Rheumatoid arthritis patients who undergo a transition from glycolysis to an oxidative based metabolism may experience a decrease in inflammation within the synovium and an improvement in the function of the immune system to contain damage (46). It should be emphasized that oxidative metabolism represents an active metabolic pathway used by macrophages to participate in specific immune functions related to containment, repair and adaptation (47). Thus, the therapeutic potential of manipulating macrophage metabolism resides not only in inhibiting glycolytic based M1 responses but also in promoting mitochondrial and lipid-oxidative capabilities that are requisite for reparative macrophage functions.

**Table 1:** Comparative summary of macrophage metabolic reprogramming in rheumatoid arthritis and coronary heart disease.

**Disease context**	**Dominant metabolic pathways**	**Major regulatory hubs**	**Predominant macrophage phenotype**	**Principal pathological consequences**
Rheumatoid arthritis (62)	Enhanced glycolysis, reduced oxidative flexibility, inflammatory metabolic bias within hypoxic synovium	Pim2 kinase, HIF-1α, GM-CSF, ERK-related signaling, AMPK imbalance, mTORC1-associated control	M1-skewed inflammatory synovial macrophages	Persistent synovitis, cytokine amplification, pannus formation, cartilage destruction, bone erosion
Rheumatoid arthritis (97)	Restored oxidative phosphorylation and fatty acid oxidation in less inflammatory or reparative states	AMPK activation, suppression of glycolytic drivers, improved mitochondrial function	M2-like or resolution-associated macrophages	Reduced inflammatory burden, improved tissue repair tendency, attenuation of structural joint damage
Coronary heart disease/atherosclerosis (37)	Increased glycolysis in plaque macrophages, inflammatory metabolic reprogramming under hypoxia and lipid stress	PI3K/AKT, HIF-1α, mTORC1, inflammatory receptor-mediated metabolic signaling	M1-skewed plaque macrophages	Plaque inflammation, necrotic core expansion, defective efferocytosis, fibrous cap weakening, plaque vulnerability
Coronary heart disease/atherosclerosis (98)	Greater oxidative phosphorylation and fatty acid oxidation in stabilizing macrophage states	AMPK-associated regulation, mitochondrial preservation, improved lipid-catabolic balance	M2-like reparative or stabilizing macrophages	Enhanced efferocytosis, reduced inflammatory escalation, greater plaque stability, lower likelihood of acute coronary events

**Table 2:** Traditional Chinese medicine monomers and compound formulas targeting macrophage metabolic reprogramming, with molecular pathways, polarization effects, disease relevance, and therapeutic implications.

**TCM intervention**	**Molecular pathways/regulatory nodes**	**Effect on macrophage polarization**	**Disease relevance**	**Therapeutic implications**
Berberine (129)	AMPK activation; downstream restraint of inflammatory metabolic signaling	Suppresses glycolysis-dominant M1 bias and favors a more oxidative, less inflammatory phenotype	Rheumatoid arthritis and coronary heart disease/atherosclerosis	May reduce inflammatory macrophage activity and support joint and vascular protection
Ginsenoside Rb1 (130)	Oxidative stress-related and immunometabolic regulatory signaling	Attenuates M1-associated inflammatory activation and supports M2-associated functional balance	Rheumatoid arthritis and coronary heart disease	May reduce inflammatory burden and improve macrophage plasticity
Quercetin acid-based strategy (including nano-delivery) (131)	ERK/HIF-1α/GLUT1 axis	Downregulates glycolysis and promotes M1-to-M2 transition	Primarily rheumatoid arthritis, with broader cross-disease relevance	Demonstrates pathway-specific macrophage metabolic reprogramming and targeted delivery potential
mTORC1/HIF-1α- or AMPK-targeting TCM formulas/monomers (132, 133)	AMPK, mTORC1, HIF-1α, PDK1, mitochondrial and nutrient-sensing pathways	Restrains M1-promoting anabolic-inflammatory metabolism while promoting oxidative, resolution-associated tendencies	Cross-disease relevance in synovitis and atherosclerotic plaque inflammation	May offer broader multi-pathway benefit where single-node interventions are insufficient

However, the functional outcome of TGF-β signaling is not unidirectional but critically depends on which Smad branch is activated in a given disease context. In atherosclerosis, activation of the Smad2/3 branch plays a protective role: TGF-β2, the predominant isoform in human carotid plaques, promotes extracellular matrix synthesis, collagen deposition, and vascular smooth muscle cell differentiation, thereby reinforcing the fibrous cap and maintaining plaque stability (48). Conversely, high-glucose conditions can redirect TGF-β1 signaling toward the Smad1/5 branch in human macrophages, upregulating the atherogenic genes HAMP and PLAUR (49), representing a vascular-deteriorating pathway. In rheumatoid arthritis, the same Smad2/3 branch—activated by TGF-β1 secreted from M2-like macrophages—can paradoxically promote myofibroblast differentiation of synovial fibroblasts and contribute to synovial fibrosis, a pathological feature of late-stage RA (50). Thus, while TGF-β is indeed associated with resolution-oriented functions in certain contexts, the precise outcome depends on the specific Smad pathway engaged and the disease microenvironment.

### Mitochondrial remodeling, redox signaling, and metabolic intermediates in macrophage fate determination

Mitochondrial function and structural changes are equally important to regulate and determine the type of metabolic control on macrophage polarization (51). Mitochondria do not simply function to produce ATP for a macrophage. They are signal-producing organelle that modulate the production of reactive oxygen species (ROS), the availability of metabolites, transcriptional responses to inflammation, and the susceptibility of apoptosis. Alteration in the structural dynamics of mitochondria, mitochondrial membrane potential, respiratory capacity, and substrate usage can all lead to an increase or decrease in the inflammatory nature of macrophages (52). Often times pro-inflammatory activation of macrophages is due to disruption of the tricarboxylic acid cycle (TCA) and alterations in mitochondrial respiration and ROS production (53). These changes may promote further inflammatory signaling. On the other hand, reparative macrophages tend to maintain intact mitochondria with flexible respiratory capabilities to support constant oxidative phosphorylation and redox balances. Redox signaling plays a critical role since ROS can be used as damaging byproducts or secondary messengers that regulate the expression of genes involved in inflammation, activate inflammasomes, and modify the responsiveness of various signaling nodes (54). Beyond structural and redox regulation, specific TCA cycle-derived immunometabolites directly influence macrophage polarization.

Itaconate is generated from cis-aconitate by IRG1 (ACOD1). In RA patients, itaconate levels in PBMCs correlate inversely with disease activity. The itaconate derivative 4-octyl itaconate (4-OI) suppresses osteoclast differentiation by inhibiting HIF-1α-mediated aerobic glycolysis, and attenuates bone erosion in arthritis mouse models (55). In atherosclerosis, itaconate produced by plaque macrophages plays a key role in diet-induced plaque resolution, and nanoparticle-based itaconate therapy reduces inflammation and induces plaque stabilization (56).

Succinate accumulates in inflammatory macrophages and stabilizes HIF-1α via prolyl hydroxylase inhibition, thereby driving IL-1β expression (57). In RA, succinate is abundantly present in synovial fluid and, through the GPR91 receptor, elicits IL-1β release from macrophages, creating a feed-forward loop of inflammatory activation. In CHD/atherosclerosis, the succinate/HIF-1α/IL-1β axis promotes endothelial inflammation and exacerbates lesion progression (58).

Lactate produced during aerobic glycolysis in macrophages regulates gene transcription via histone lysine lactylation, a recently discovered epigenetic modification that modulates macrophage polarization and inflammatory responses (59). In atherosclerosis, MCT4-mediated histone H3 lysine 18 lactylation (H3K18la) is closely involved in the stage-specific local repair process during M1-to-M2 transition; MCT4 deficiency activates reparative genes and protects from atherosclerosis, while histone methylation and acetylation are not involved in this process (60).

Mechanistic support for the claim that mitochondrial integrity governs reparative macrophage survival has recently emerged. SerpinB2 (plasminogen activator inhibitor type 2) promotes adipose tissue-resident macrophage survival by regulating mitochondrial oxidative phosphorylation (OXPHOS) and preventing the release of pro-apoptotic cytochrome c from mitochondria into the cytoplasm via antioxidant glutathione production (61). Chronic inflammation diminishes SerpinB2 expression, leading to the decline of this macrophage subset. Notably, supplementation with N-acetylcysteine, a glutathione precursor, restores resident macrophage survival, decreases adipocyte size, and improves glucose tolerance and insulin sensitivity. These findings provide direct molecular evidence that mitochondrial OXPHOS capacity and integrity are causally required for the survival of tissue-resident macrophages under inflammatory stress, supporting the conceptual framework of the present review.

### Core signaling hubs regulating macrophage metabolic reprogramming: Hif-1α, AMPK, mTORC1, and Pi3K/Akt

**Figure 1:**
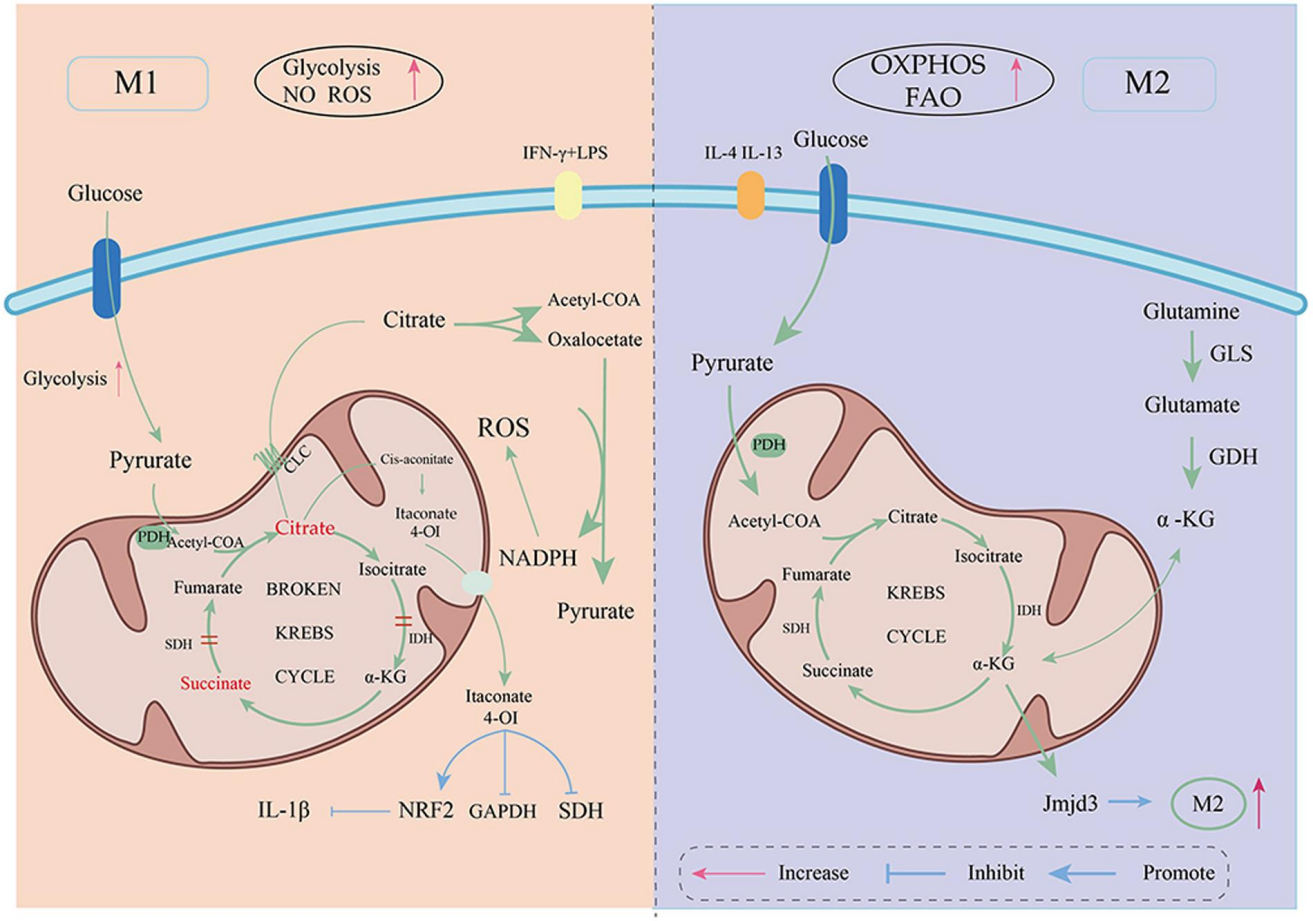
The metabolic differences between M1 and M2 macrophages (67).

The switch from glycolysis-driven inflammatory macrophages (M1) to oxidative repair macrophages (M2), is regulated by a group of central signaling units; through integration of various forms of environmental stress, the nutritional state of the cell, inflammatory signals, and intracellular energy balance (62). Among these groups, HIF-1α, AMPK, mTORC1, and PI3K/AKT are especially important (63). As a master regulator of adaptation to hypoxia and pseudo-hypoxia, HIF-1α drives glycolytic metabolism by regulating glucose transporters and glycolytic enzyme levels, and supports the expression of genes involved in inflammation. When macrophages are exposed to hypoxia, cytokines or inflammatory tissue stress, HIF-1α plays a significant role in stabilizing the M1-like phenotype of macrophages by linking environmental stresses to glycolytic reprogramming (64). Conversely, the cellular energy sensing mechanism AMPK acts as a counterpoint to this process. As an energetic sensor, it is activated during times of reduced energy availability and promotes catabolism, mitochondrial biogenesis, and fatty acid oxidation (65). Moreover, it limits excessive anabolic and inflammatory signaling. Therefore, its activation is typically seen with the transition towards oxidative metabolism and a less inflammatory macrophage phenotype. mTORC1 regulates anabolic metabolism, protein synthesis, and immune response based on the availability of nutrients and growth factors. It has been shown to promote glycolytic and inflammatory programming in macrophages (66). Finally, PI3K/AKT signaling integrates growth factor/ inflammatory signals with glucose metabolism/survival pathways, leading to the activation of subsequent metabolically active proteins (21). Importantly, none of these nodes work independently but rather form an interdependent regulatory network in which hypoxia, nutrient abundance or lack thereof, cytokine exposure, and tissue stress can be converted into stable metabolic decision making. Furthermore, given that the same regulatory mechanisms are present within both rheumatoid arthritis and atherosclerotic plaque biology although their tissue microenvironments vary significantly, understanding how these nodes regulate macrophage metabolic processes and outputs will provide a rational basis for targeting interventions such as traditional Chinese medicine monomers and formulas that have demonstrated effects at the very same metabolic control points. To summarize the relationships outlined in this section, Figure 1 depicts the integrated logic of macrophage immunometabolism, highlighting the differences between glycolysis-dominant M1 polarization and oxidative phosphorylation/fatty acid oxidation-dominant M2 polarization (67).

## Metabolic reprogramming and macrophage polarization in rheumatoid arthritis

### Synovial macrophages as immunometabolic drivers of rheumatoid inflammation

The primary effector macrophage within the rheumatoid arthritic (RA) joint is likely the synovial macrophage as these are found centrally at the point where immune responses lead to metabolic changes and structural damage (68). In normal conditions, macrophages play roles in monitoring for potential pathogens, clearing cellular debris, and maintaining homeostasis. However, with RA the synovium becomes a chronically inflamed and metabolically challenged environment due to factors such as hypoxia, overabundance of cytokines, alteration in nutrient availability, increased white blood cell invasion of the joint space, and increase of fibroblast-like synoviocytes (FLS) (69). At this point, rather than simply responding to the ongoing inflammation of the RA joint, synovial macrophages take on an active organizational role. The organization of inflammation is mediated through production of TNFα, IL-1β, IL-6, chemokines, proteolytic enzymes capable of degrading connective tissue matrices, and lipid derived inflammatory signaling molecules that facilitate continued recruitment of white blood cells into the inflamed area, enhance stromal cell activation and perpetuate local immune system presence (70). Furthermore, the level of participation of synovial macrophages in chronic inflammatory disease may be enhanced by virtue of the fact that these cells have been shown to exhibit metabolic flexibility (71). Upon adaptation to the RA microenvironment, synovial macrophages undergo metabolic reprogramming to develop a phenotypic profile that supports a chronic inflammatory response (72).

### Pim2 kinase, Pgk1, Pdha1, and enzymatic control of rheumatoid macrophage metabolism

Although rheumatoid macrophages have a glycolytic and inflammatory phenotype based on both external pressures (environmental) and internal regulators that link metabolic enzyme regulation to macrophage polarization, their intracellular regulators directly regulate how the environmental pressure influences macrophage function. One of the most interesting regulators identified recently is the Pim2 protein kinase, which acts as an intracellular regulator linking inflammatory signals to select metabolic pathways used by rheumatoid arthritis synovial macrophages (15). In terms of mechanism, Pim2 regulates glycolytic activity and supports M1-like macrophage polarization via phosphorylation of metabolic enzymes including phosphoglycerate kinase 1 (PGK-1) and pyruvate dehydrogenase E1 alpha 1 (PDHA1) (10). These mechanisms are critical as they demonstrate that macrophage polarization in rheumatoid arthritis does not occur exclusively at the level of transcription factor expression or cytokine receptor activation; instead, macrophage polarization occurs through posttranslational modification of key metabolic components. PGK-1 plays a key role in glycolysis, converting substrate flux into both energy and biosynthetic precursors necessary to support inflammatory function (73).

Notably, while canonical rate-limiting glycolytic enzymes such as PFK1, HK2, and PKM2 are ubiquitously expressed and primarily respond to cellular energy demands, they lack the disease-specific upregulation that characterizes RA pathology. In contrast, Pim2 and its downstream target PGK1 represent a distinct regulatory hierarchy: Pim2 functions as an upstream orchestrator that directly phosphorylates PGK1-S203, PDHA1-S300, and PFKFB2-S466, thereby driving glycolytic reprogramming in inflammatory macrophages (15). This hierarchical positioning enables Pim2 to systemically amplify glycolytic flux beyond conventional energy-sensing regulation, a feature particularly relevant under the sustained inflammatory stress of the RA synovium. Moreover, both Pim2 and PGK1 exhibit RA-specific upregulation: Pim2 expression is markedly elevated in RA macrophages and its inhibition reverses M1/M2 imbalance, while PGK1 levels are increased in RA synovial tissues and serum, correlating with disease activity and pro-inflammatory cytokine production (74). Mechanistically, PGK1 has been identified as a key effector enzyme within the glycolysis-HIF-1α-IL-1β positive feedback loop that amplifies synovial inflammation in RA (75). Taken together, Pim2 and PGK1 are disease-amplifying nodes rather than mere housekeeping metabolic enzymes, making them attractive and selective therapeutic targets for restoring immunometabolic balance in RA.

### GM-CSF-induced high-metabolic macrophage phenotypes in rheumatoid arthritis

In rheumatoid arthritis (RA), granulocyte-macrophage colony-stimulating factor (GM-CSF) has emerged as a key driver of synovial macrophage metabolic reprogramming and persistent inflammation (76). Mechanistic studies have revealed that GM-CSF profoundly alters macrophage bioenergetics. Specifically, GM-CSF increases the extent of lipopolysaccharide (LPS)-induced acute glycolysis in macrophages, and pharmacological inhibition of glycolysis using 2-deoxyglucose abolishes GM-CSF-mediated upregulation of tumor necrosis factor-α (TNF-α), interleukin (IL)-1β, IL-6 and IL-12p70 synthesis upon LPS stimulation (77). This glycolytic shift is essential for the pro-inflammatory functions of GM-CSF-primed macrophages. Beyond metabolic changes, GM-CSF also shapes the chemokine profile of RA macrophages. Among the chemokines upregulated in RA monocyte-derived macrophages, CCL22 is the most prominent; it promotes CD4⁺ T cell migration and skews differentiation toward Th1 and Th17 subsets, thereby amplifying the inflammatory cascade within the rheumatoid joint (78). The translational relevance of targeting this pathway has been demonstrated in clinical studies. Notably, blockade of the GM-CSF pathway using the anti-GM-CSF receptor-α monoclonal antibody mavrilimumab significantly decreases RA disease activity, with clinically meaningful responses observed as early as one week after treatment initiation (79). Cumulatively, these findings position GM-CSF not only as an immune signal but as a central metabolic organizer that links cytokine-driven inflammation with the bioenergetic demands of pathogenic macrophages in RA.

## Metabolic reprogramming and macrophage polarization in atherosclerosis and coronary heart disease

### Atherosclerosis as the inflammatory and metabolic substrate of coronary heart disease

The coronary artery disease is primarily due to the development of atherosclerosis, which is a long-term inflammatory and metabolic disorder of the vascular walls (80). Atherosclerotic plaque forms in an active biological setting created through the effects of impaired endothelial function, lipoprotein deposition and modification, abnormal flow characteristics (shear stress), oxidative damage, sterile inflammation, and inappropriate activation of immune-cells (17). Monocytes migrate to the vessel intima and then differentiate into macrophages that very quickly assume a role as critical regulatory cells in determining the progression of atherosclerotic lesions (81). Macrophages are not simply clearing agents of lipid debris. They can be considered to be metabolically responsive inflammatory cells whose functional phenotype is determined by the plaque microenvironment, and their activity dictates whether a lesion will remain relatively stable or progress towards instability (82). The atherosclerotic plaque creates a unique metabolic environment with pressure on the macrophage to include low oxygen levels, high cholesterol content, excessive oxidative stress, dysfunctional efferocytosis, and abundant inflammatory signals (83). As a result, these environments induce metabolic remodeling in macrophages and affect the balance between pro-inflammatory/tissue damaging processes and anti-inflammatory/stabilizing processes. Therefore, atherosclerosis can be considered to be both an inflammatory condition and an immunometabolic disease process. Consequently, understanding the metabolic regulation of macrophages in the atherosclerotic plaque is critical for understanding why coronary lesions evolve from sub-clinical alterations in arterial structure to clinically unstable plaques capable of inducing acute ischemic episodes.

### PI3K/Akt-mediated metabolic control of macrophage activation in atherosclerotic lesions

PI3K/AKT has a unique role among the many signaling mechanisms that modulate macrophage metabolism in atherosclerosis due to its ability to translate external inflammatory and growth stimuli into internal metabolic programs (84). The activation of PI3K/AKT leads to enhanced glucose uptake, elevated glycolytic rates, support of cellular survival functions and enhancement of anabolic functions that are consistent with the type of activation seen in inflammatory macrophages (85). These signals may originate from a variety of sources within the atherosclerotic plaque such as modified lipoproteins (e.g., oxidized LDL), cytokines, growth factors and various inflammatory receptors. Upon activation, PI3K/AKT maintains a metabolic profile that is conducive to M1-like activation which supports high levels of pro-inflammatory mediator production and sustains chronic inflammation in the plaque (86). As such, PI3K/AKT serves an integrating function. Rather than being simply an inflammatory “on/off” switch, PI3K/AKT integrates plaque-related environmental stresses into metabolic behaviors that determine macrophage identity and the final pathology of the lesion (87). Furthermore, through its effects on glucose metabolism and related signaling networks, PI3K/AKT will reinforce the glycolytic preference that is characteristic of inflammatory plaque macrophages. These changes lead to increased cytokine secretion; decreased ability to resolve inflammation; continuous recruitment of leukocytes; and progressive structural deterioration of the plaque.

### OXPHOS, fatty acid oxidation, and M2-associated mechanisms of plaque stabilization

Oxidative phosphorylation and faty acid oxidation are associated with macrophage function that is more like that of resolving inflammation than producing it; these functions enhance plateau stabilization, tissue adaptation and plateau resolution (41). Metabolic efficiency (ATP generation), mitochondrial integrity, and metabolic flexibility are all advantageous for long term homeostatic tasks such as efferoctisis (cell clearance) of apoptotic cells (88). Also they reduce the amount of excessive inflammatory cytokine production by limiting their release from plaque macrophages. These funtional importance of oxphos/FAO macrophage states are mostly observed in the more stable plaques where cell clearance and containment of debris reduce necrotic core expansion and help preserve lesion architecture (89). Additionally, oxidative phosphorylation supports a repariative macrophage phenotype by promoting catabolic efficiency and reduction in reliance on rapid glycolytic flux to sustain inflammation (90). Thus, oxidative phosphorylation and FAO associated macrophage states are generally considered to serve as biological counterweights to plaque inflammation. However, the finding that macrophage *de novo* fatty acid synthesis via ACLY/FASN drives pathogenic fibroblast expansion and fibrosis after MI raises the possibility that in certain contexts—particularly where tissue fibrosis predominates over inflammation—activation of the fatty acid synthesis pathway may be maladaptive (45). This suggests that the metabolic control of macrophage function in cardiovascular disease is more nuanced than a simple binary of glycolysis = bad vs. FAO = good, and that the pathogenic versus protective outcome likely depends on the specific disease stage, tissue microenvironment, and the balance between FAO and *de novo* lipogenesis.

### Metabolic determinants of plaque vulnerability and coronary outcomes

Plaque vulnerability is significantly impacted when considering macrophage metabolic reprogramming and how it correlates with acute coronary events. These events (plaque rupture/erosion, thrombosis, and subsequent ischemia) are not simply related to the degree of luminal narrowing, but rather represent a functional state of the lesion itself, specifically the relative contribution of inflammation-induced injury vs. structural stability (91). Macrophages have a significant role in determining this functional state due to their influence on cytokine production, protease release, efferocytosis efficiency, lipid processing, oxidative stress, and interactions with vascular smooth muscle cells and the extracellular matrix (92).

Macrophages with glycolysis-dominant metabolism and skewed towards an M1 phenotype are associated with thinning of the fibrous cap and increase in the area of the necrotic core, resulting in an increased likelihood of plaque rupture and increased inflammatory susceptibility (93). Additionally, these macrophages further exacerbate the intraplaque environment by increasing apoptosis and impeding effective clearance of apoptotic material. Conversely, macrophages utilizing oxidative phosphorylation and β-oxidation for energy metabolism tend to support maintaining the integrity of the plaque structure, facilitating removal of dead cellular debris and preserving plaque architecture (94). Therefore, the proportion of each metabolic profile determines if atherosclerotic lesions remain relatively stable or evolve into “high-risk” plaques which can precipitate myocardial ischemia.

The molecular basis linking defective efferocytosis in vulnerable plaques to necrotic core expansion is now understood at the epigenetic level. Recent studies demonstrate that RBPJ (Recombination Signal Binding Protein for Immunoglobulin Kappa J Region), a transcription factor involved in canonical Notch signaling, directly controls macrophage efferocytic capacity in atherosclerotic plaques. CUT&RUN sequencing revealed that RBPJ selectively diminishes the repressive H3K9me3 heterochromatin mark on the promoters of Stard13 and Arsg, thereby upregulating these genes (95). Stard13 promotes efferocytosis by inhibiting Rho and activating RAC GTPases, facilitating actin polymerization and apoptotic cell uptake. Conversely, inhibition of γ-secretase or genetic deletion of RBPJ significantly reduces efferocytosis. Endothelial-specific deletion of RBPJ in ApoE⁻/⁻ mice results in reduced atherosclerosis, correlating with decreased leukocyte rolling and lower macrophage content in the vascular wall (96). Collectively, these findings demonstrate that impaired efferocytosis in vulnerable plaques reflects a specific epigenetic lesion—H3K9me3-mediated silencing of Stard13 and Arsg—which can be therapeutically restored by targeting SUV39H1/H2 methyltransferases or upstream RBPJ signaling. Thus, coronary event risk is determined by factors other than just plaque size or degree of stenosis including the immunomodulatory properties of lesional macrophages. The table that follows summarizes this comparison by outlining the major metabolic pathways, regulatory hubs, macrophage phenotypes, and pathological consequences that characterize macrophage reprogramming in rheumatoid arthritis and coronary heart disease.

## Shared mechanistic nodes linking rheumatoid arthritis and coronary heart disease

### Convergent immunometabolic architecture across synovial inflammation and atherogenesis

Although RA and CHD present clinically differently, they are supported by an identical immunometabolic structure that macrophages play as primary processors of inflammatory signals to create tissue injury. The RA synovium is a chronically active site due to low oxygen levels (hypoxia), excessive cytokines and white blood cells, changed nutrient supplies, increased number of stroma cells (99). Whereas the CHD arterial intimal layer develops as a metabolically stressed inflammatory lesion with dysfunctional endothelium, accumulated modified lipid particles, oxidative stress, dead cells and ineffective efferocytosis (100). Although the two lesions are different in their sites within the body and triggers for activation, both lesions provide macrophage populations with similar selective pressures. Both RA and CHD provide macrophages that undergo glucose metabolism, maintain an inflammatory phenotype and produce local inflammatory mediators including cytokines, chemokines, proteases and oxidants that lead to further disease progression (4). In both diseases, macrophages have a pathologic role that extends beyond traditional roles of immune cells.

### Shared regulatory checkpoints: Hif-1α, AMPK, mTORC1, Pi3K/Akt, and mitochondrial signaling

The connection between Rheumatoid Arthritis (RA) and Coronary Heart Disease (CHD) is further illustrated when considering the cell-type regulatory checkpoints for RA and CHD at the cellular level. Signaling pathways including; HIF-1α, AMPK, mTORC1, PI3K/AKT and mitochondrial signaling create a complex web that determines if macrophages will exhibit glycolytic inflammatory responses or oxidative repair mechanisms (9). Specifically, HIF-1α plays a major role in both diseases due to hypoxia or pseudo-hypoxia found in most diseased tissues leading to its stabilization; stabilized HIF-1α leads to increased glucose uptake, upregulation of glycolytic enzymes and increased inflammatory response from macrophages (101, 102). For example, HIF-1α's stabilization supports chronic M1 like macrophage populations in RA synovial tissue (103). Similar to RA, activation of HIF-1α also promotes glycolysis driven inflammatory macrophage behaviors in atherosclerotic plaques during hypoxia, lipid stress, and plaque expansion (104). Conversely, AMPK acts as a metabolic counter regulator supporting energy efficiency, mitochondrial function, and fatty acid oxidation; inhibiting excessive anabolic and inflammatory signaling (105). Therefore, loss of AMPK dominant control results in an inflammatory biased response in both disease states; conversely, restoring AMPK signaling has the potential to be beneficial in creating macrophage phenotypes supportive of resolution processes. mTORC1 and PI3K/AKT integrate signals regarding nutrient availability, growth factor signals, and inflammatory receptor inputs with enhancement of glycolysis and anabolism-based immune responses (106). Ultimately, mitochondrial signaling provides another layer of regulation over the respiratory flexibility, redox state, ROS production and metabolite-dependent signaling. These checkpoints provide a common framework used by various tissue microenvironments to produce similar macrophage outcomes. Thus, it is expected that the same pathways will continue to emerge across studies examining RA synovitis and atherosclerosis plaque biology despite differences in upstream stimuli.

### Rheumatoid arthritis as a systemic accelerator of coronary heart disease

Epidemiological studies have consistently demonstrated an association between rheumatoid arthritis (RA) and an increased risk of coronary artery disease (CAD) (82). While causality remains to be definitively established, accumulating evidence suggests that RA may promote CAD development and/or progression through chronic systemic inflammatory “spillover” into the coronary circulation, as well as through long-term disturbances to the host's immunometabolism (107). Chronic inflammatory processes within the synovium do not remain localized to the joint. Instead, they contribute to an extracellular environment characterized by elevated levels of cytokines; acute phase reactants; oxidative stress indicators; abnormalities in lipid processing; and defects in endothelial function (108). As such these systemic changes promote atherosclerosis and potentially reduce the “threshold” at which plaques begin to develop inflammation and evolve towards rupture. Therefore, based on current evidence, RA may represent a chronic extra-articular contributor to coronary artery disease risk. This is hypothesized to occur not simply because of widespread inflammation, but rather because the disease places pressure on the vascular compartment through the exportation of metabolic-inflammatory signals (109). Immune cells present in the circulation are conditionally predisposed to exhibit inflammatory phenotypes; the integrity of vascular endothelium is compromised; and the likelihood of atherosclerotic macrophages developing a similar glycolytic phenotype and adopting the same pro-inflammatory programs as those resident within the RA synovium is enhanced (110). As such, a plausible biologic rationale has been proposed to explain how RA disease activity might contribute to accelerated plaque development and, consequently, an increased risk of coronary events. However, direct evidence for this causal chain in humans remains limited, and the proposed mechanisms are largely derived from animal models and *in vitro* studies that require further validation in patient populations. Given the fact that the cardiovascular burden associated with RA is frequently greater than would be predicted based on traditional risk factors alone, this conceptualization provides particular relevance. Additionally, given that there exists a common macropahge centered immunometabolic basis for RA related excess cardiovascular risk; this conceptualization supports the potential dual benefit to both articular and cardiovascular outcomes resulting from improved systemic control over inflammatory metabolism.

### Conceptual model of Ra–Chd comorbidity through macrophage-centered metabolic dysregulation

Based on the associative and mechanistic evidence reviewed above, we propose a hypothetical conceptual model in which macrophage-centered metabolic dysregulation links rheumatoid arthritis (RA) and coronary heart disease (CHD) (111). It is important to emphasize that this model is speculative and intended to generate testable hypotheses, rather than to assert a proven causal pathway. The proposed model suggests that RA-driven chronic inflammatory stress activates synovial macrophages towards an M1-like activation profile with a preference for glycolytic metabolism via known pathways of HIF-1α, PI3K/AKT, mTORC1, and other specific RA-regulatory nodes (i.e., Pim2). The activated macrophages lead to localized tissue damage; however, they also sustain systemic inflammatory signals to modify vascular biology and immunocellular behaviors (111). As macrophages within the arterial wall become exposed to endothelial dysfunction, modified lipoproteins, oxidative stress, and various inflammatory mediators, they undergo a similar metabolic transition toward glycolysis with increased inflammatory potential and decreased repair capabilities. Both sets of macrophages have been demonstrated to lack sufficient AMPK-mediated restriction, have impaired mitochondria function and weakened oxidative program resulting in their inability to convert to pro-resolve states (112). Therefore, it is hypothesized that these two diseases may reinforce each other through a common macrophage-centered immunometabolic axis. This hypothetical framework requires direct testing in longitudinal human cohort studies and interventional trials. Such a model will provide valuable insight for developing therapy strategies that restore balance to macrophage metabolism to simultaneously treat the RA activity and cardiovascular risk. Additionally, the proposed model provides support for incorporating Traditional Chinese Medicine intervention methods that target the HIF-1α pathway, the AMPK pathway, the mTORC1 pathway, the PDK1 pathway and others into a larger macrophage-centric therapeutic strategy.

## Traditional Chinese medicine intervention strategies targeting macrophage metabolic reprogramming

The use of traditional chinese medicine (TCM) has become increasingly popular for treatment of both rheumatoid arthritis and coronary heart disease (113, 114). However, it is important to note that the majority of evidence discussed in this section is derived from *in vitro* studies, animal models, or small-scale preclinical experiments, and clinical translation remains at an early stage. TCM offers a pharmacologically-rich source of bioactive compounds that may have the ability to target a variety of inflammatory and metabolic pathways simultaneously, but robust clinical validation is currently lacking. This study was developed using the premise that TCM can potentially modulate the immunometabolic machine determining macrophage function. Macrophages exist in either a pro-inflammatory M1 or anti-inflammatory M2 phenotypic state. Glycolytic-dominant M1 macrophages contribute to the chronic inflammation seen in the joint of patients suffering from Rheumatoid Arthritis (RA), as well as the plaque progression in atherosclerosis (115). Oxidative phosphorylation/fatty acid oxidation-associated M2 macrophages promote the resolution of inflammation and preservation of tissue integrity (116). Therefore, developing therapies targeting the regulation of macrophage metabolism may provide significant opportunities for translation. Many components of TCM, as well as compound combinations, appear to be effective at regulating the signaling pathways involved in the connection between metabolism and inflammatory macrophage function, including PDK1/PI3K/AKT/mTORC1, AMPK, HIF-1α and others.

Berberine has been shown to activate AMPK in preclinical models (23). Based on these findings, it is hypothesized that AMPK activation could cause changes in macrophage behavior … However, direct evidence that berberine exerts this effect in RA or CHD patients remains limited (117). As such, activating AMPK could potentially cause changes in macrophage behavior by moving them from being primarily glycolytic cells producing pro-inflammatory responses towards being more metabolically balanced and resolving inflammatory responses. Both RA and CHD have associations with reduced levels of functional AMPK that contribute to persistent inflammatory macrophage behaviors (65). Ginsenoside Rb1 is another compound that has emerged as an immunometabolically relevant pathway-specific compound. The effects of ginsenoside Rb1 appear to affect multiple aspects of cellular biology but there is some indication that it can decrease inflammatory stress, modify oxidative damage and reorient macrophage phenotypes toward less persistent M1 polarized states (118). These types of compounds are especially important because they represent potential ways to “re-program” the metabolic environment in which macrophages interpret their inflammatory cues rather than suppressing inflammation per se. Nevertheless, most studies to date have been conducted in isolated cell systems or animal models, and whether these effects translate to human disease is not yet established.

Traditional chinese medicinal (TCM) therapy that utilizes the HIF-1a/PDK1 axis for regulation has an added level of relevance due to its ability to possibly interact with many different points on the signaling cascade. The use of TCM formulas that can modulate the HIF-1 alpha/PDK1 axis would have great value as HIF-1 alpha plays a large role in the glycolytic adaptive response of inflammatory macrophages and PDK1 limits the amount of pyruvate entering mitochondria from glycolysis (119). Thus, the modulation of these two proteins may allow for the reduction of the metabolic stability of M1 like macrophages and allow for an increase in oxidative flexibility. Also, other TCM formulas may work via the AMPK, mTORC1, or PI3K/AKT related signaling pathways which could influence nutrient sensing, inflammatory metabolism and cell energy allocation in a system pharmacological fashion (120). Due to this potential for multi-targeting, TCM formulas may be especially beneficial in cases of patients who suffer from rheumatoid arthritis and concomitant coronary heart disease. In this population, joint inflammation and vascular dysfunction along with elevated levels of systemic cytokines and alterations in lipid metabolism all converge to create a situation where a therapeutic strategy based solely on blocking one path way will not be sufficient.

There are other advances in translation with respect to the use of nano-delivery systems for targeted, enhanced stability, and enhanced tissue penetration of various compounds derived from traditional chinese medicine (121). The reason this is an important area of study is that the effectiveness of immunomodulatory compounds as therapeutics is based upon both their ability to influence specific pathways (e.g., metabolic) and their successful delivery to areas where inflammation occurs (microenvironments) (122). Thus, nano-delivery systems for anti-inflammatory agents such as those used to treat rheumatoid arthritis could potentially deliver drugs directly to inflamed tissues where macrophage metabolism is altered due to low oxygen levels and/or increased metabolic stress. Likewise, optimizing drug delivery via nano-delivery systems may provide a method of delivering drugs specifically to plaque associated macrophages in atherosclerosis while minimizing the non-specific effects of the drug throughout the body.

Clinical evidence for TCM compounds remains heterogeneous. Berberine, known for its poor oral bioavailability, has been formulated as the derivative HTD1801 (berberine ursodeoxycholate), which enhances absorption. A 2025 phase 2 RCT in T2DM patients demonstrated significant HbA1c reductions, supporting its potential for metabolic modulation (123). Emodin has been evaluated in collagen-induced arthritis rat models and shown to alleviate synovitis via modulation of the HIF-1α/NLRP3 inflammasome pathway, downregulating IL-6 and IL-1β; however, human bioavailability and dose-response data are lacking, and the current literature remains primarily preclinical (124). For Tripterygium wilfordii Hook F, a 24-week multicenter RCT (*n* = 64) reported non-inferiority of TwHF combined with adalimumab to adalimumab plus methotrexate, and a separate RCT (*n* = 62 completers) showed an ACR20 response of 65.0% with TwHF versus 32.8% with sulfasalazine at 24 weeks (125). Nevertheless, concerns over hepatotoxicity, reproductive toxicity, and inconsistent formulations hinder clinical translation. Quercetin has been formulated into microenvironmental enzyme-responsive methotrexate-modified micelles for targeted RA therapy, demonstrating joint-swelling reduction in CIA rats, but human data remain absent (126). Finally, a 2025 meta-analysis (42 RCTs, 4,654 patients) demonstrated that sodium tanshinone IIA sulfonate injection reduces IL-6, TNF-α, ICAM-1, and MCP-1 levels in atherosclerosis patients, supporting its clinical anti-inflammatory utility (127). Collectively, these six compounds provide preliminary evidence linking TCM to immunometabolic modulation, but the overall translational readiness remains low. Future research must prioritize standardized formulations, improved bioavailability, and rigorous RCTs with patient-relevant outcomes in RA-CHD populations.

Traditional Chinese Medicine (TCM) has an important role as an integral part of therapy for patients with rheumatoid arthritis (RA), and concomitant coronary heart disease (CHD) (113). The potential greatest future use of TCM is likely going to be through integrating therapeutic designs into modern medicine (128). From a macrophage centered perspective, interventions are selected based on controlling symptoms; however, it also should include modulating the metabolic pathways involved in the inflammatory response to restore balance across both the vascular and synovial tissues. Therefore, TCM monomers, formulas and delivery systems could potentially work in conjunction with conventional treatments to target the metabolically active infrastructure of chronic inflammation. However, to realize this translational potential, there needs to be increased mechanistic validation, improved standardized formulation quality, and additional research demonstrating how modifying specific pathways result in clinically meaningful outcomes. Despite these promising preclinical findings, the current evidence base remains preliminary. As acknowledged above, most studies are *in vitro* or animal-based, and clinical data are sparse. A more accurate conclusion is that TCM represents a biologically plausible, but as yet unproven, approach to modulating macrophage immunometabolism in the context of RA-CHD comorbidity. Rigorous mechanistic studies, standardized formulations, and well-designed clinical trials are required before any claim of translational readiness can be made.

### Interfering with lactate-driven immunometabolic pathways: a new horizon for Tcm

Beyond its role as a glycolytic byproduct, lactate has emerged as a central signaling molecule and epigenetic substrate that links macrophage metabolism to disease pathogenesis in both RA and CHD. In primary human macrophages, high glucose stimulation increases lactate production and activates the atherogenic TGFβ-Smad1/5 pathway, upregulating the expression of HAMP and PLAUR—two genes closely associated with atherosclerotic lesion progression (49). This finding provides a direct mechanistic explanation for how lactate accumulation in the diabetic milieu — or, by extension, in the chronically inflamed synovial environment of RA — may accelerate coronary heart disease. In RA, lactate exerts context-dependent effects: at early stages, it promotes M1-like polarization and fuels synovial inflammation; at later stages, it facilitates M2-like polarization and tissue repair via histone lactylation. Recent studies have further established that lactate exacerbates RA through activation of the cGAS-STING signaling pathway in macrophages (134).

Importantly, several active components of traditional Chinese medicine have been shown to modulate lactate metabolism and lactylation. Berberine reduces lactate production via AMPK activation and ameliorates collagen-induced arthritis by switching glycolytic reprogramming. Ginsenoside compound K, baicalin, and cordycepin have also been reported to decrease lactate levels and suppress lactylation-driven inflammation in preclinical models of metabolic and inflammatory diseases (135). Although direct evidence in RA-CHD comorbid populations is still lacking, these observations suggest that TCM-based interventions targeting the lactate-centric metabolic-epigenetic axis may offer a novel strategy for restoring immunometabolic homeostasis. Future studies should specifically examine whether berberine, ginsenosides, or TCM formulas can interfere with the lactate-TGFβ-Smad1/5 pathway in atherosclerotic plaques, and whether such interference translates into reduced cardiovascular risk in RA patients.

## Future directions

Research into Rheumatoid Arthritis (RA) and Coronary Heart Disease (CHD), while interesting for comparison purposes, should focus on creating an integrated Immunometabolic Framework based upon Macrophage Plasticity. The first step will be to determine how Metabolically-Active States of Macrophages transition through Time as they relate to RA and CHD. Specifically, researchers are looking to study individuals with RA who develop Cardiovascular Complications. Currently, studies regarding RA and CHD are either cross-sectional or disease specific. These limitations create challenges for researchers wishing to understand whether Glycolytic Macrophage Bias in RA Synovial Tissue and Atherosclerotic Plaques represent a Shared Initial Program; a Converging Downstream Response; or a Dynamic Sequence that evolves based on Systemic Inflammatory Burden. As such, researchers would greatly benefit from longitudinal studies that follow changes in metabolic signaling, macrophage phenotypes, inflammatory mediators, and clinical outcomes in both Articular Compartments (Joints) and Cardiovascular Compartments. Ultimately, this type of research may help investigators determine if RA-induced Inflammation can directly prime circulating Monocytes/Tissue Macrophages toward a Coronary Risk Phenotype. Additionally, identifying those Stages where Intervention is likely to be most successful represents a critical goal of future research.

The field requires a strong mechanistic validation for all regulatory check-points discussed in this review (i.e., HIF-1α, AMPK, mTORC1, PI3K/AKT, etc. along with enzymatic regulation of glycolysis), although each of these pathways have been implicated in multiple studies as being involved in regulating experimental outcomes, their hierarchical relationships and disease stage dependency and therapeutic tractability has not been resolved. Additional study will be required to understand how Pim2 kinase, PGK1, PDHA1 and GM-CSF associated metabolic programs integrate with conventional signal transduction pathways in RA. Similarly, further study is needed to establish what role metabolic regulators play in influencing the heterogeneity of plaque macrophages in coronary artery disease, efferocytosis, fibrous cap stability and progression to clinically relevant lesions. Comparative mechanistic studies across both diseases may provide information on whether certain pathways represent common therapeutic targets or if they are organ specific.

A second area of focus will be developing translational models guided by biomarkers. Although reprogramming of macrophage metabolism has a strong mechanistic rationale it does not at present lend itself well to measurement in routine clinical care. The next step will therefore involve identifying biomarker panels which indicate an imbalance of macrophage metabolic bias and enable the stratification of patients based on their predominant pathophysiological mechanism. These panels may include a combination of circulating inflammatory mediators, circulating metabolic enzymes, lipid related signals, imaging related markers or cellular immune related markers. Biomarker-guided stratification in RA patients who have comorbid CVD could aid in identifying those patients where macrophage centered metabolic intervention(s) will result in maximum dual benefits for both joint inflammation and vascular health. Therefore, this will transition the field away from general inflammatory suppression toward precision oriented immunometabolic therapies.

A comparable translational commitment exists for traditional Chinese medicine. There are numerous monomers and formulations that have been demonstrated to modulate macrophage metabolic pathways; however, the current state-of-the-art lacks uniformity regarding mechanistic detail, formulation consistency, dosing protocols, and disease-specific application. The next step will be to go beyond general anti-inflammatory claims. It is necessary to conduct studies to determine whether there are traditional Chinese medicine-based interventions that specifically can modulate the macrophage's glycolytic pathway, restore its oxidative capacity, or modify other established regulatory checkpoints (i.e., HIF-1α, AMPK, mTORC1, PDK1, and PI3K/AKT). Well-designed animal studies and cellular studies will need to precede the design of high-quality clinical studies conducted within well-defined patient populations (i.e., patients with rheumatoid arthritis who also have a comorbid coronary condition). In addition to standardizing the chemical composition of compounds used in these studies, it will be critical to develop standard pharmacokinetic profiles and quality assurance/quality control programs for these compounds before translating traditional Chinese medicine into an effective macrophage-targeted therapy as opposed to merely maintaining a conceptual basis without clinical relevance.

Nano-delivery and targeted delivery systems will provide additional opportunities to further develop macrophage metabolic therapy. The successful implementation of this approach requires both an appropriate pathway as well as localized delivery into inflamed tissue. Therefore, we believe it is essential to assess methods which increase localization within plaques/synovia; enhance intracellular delivery of compounds into macrophages; decrease non-targeted exposure and/or utilize controlled drug delivery in stressed metabolically compromised environments. In addition, these approaches would likely have value for compounds that exhibit bioactivity, yet suffer from poor water solubility, poor chemical stability, or poor diffusion through tissues.

Ultimately, future clinical trials should utilize a dual-disease approach as opposed to individually researching rheumatoid arthritis (RA) and coronary heart disease (CHD). Because an increasing body of evidence has established that both diseases have macrophage-centered metabolic dysfunction; therefore, interventional studies should be designed to evaluate how these interventions can modify joint inflammation and vascular pathology simultaneously. In this way, combination therapies utilizing conventional immune-modulating agents, metabolic regulators, compounds derived from traditional Chinese medicines and novel drug-delivery technologies could be tested together in a single mechanistic framework. Ultimately, it is hoped that a transition will occur from managing symptoms related to RA and CHD separately, towards a system level management of RA-CHD comorbidities where macrophage immunometabolic functions are used as the primary therapeutic axis for preventing structural joint destruction, stabilization of vascular lesions, and reduction of the cumulative impact of RA-CHD comorbidities.

## Conclusion

The results from this review support the hypothesis that macrophage metabolic changes may serve as a key connection between RA and CHD, extending beyond a mere biochemical side effect of inflammation. However, the majority of evidence reviewed is associative in nature, and the proposed causal relationships remain to be confirmed by prospective studies and experimental models that directly test the directionality of these interactions. It is important to note that much of the evidence establishing the relationship between glycolysis and M1 polarization remains associative in nature, and the precise directionality of this relationship—whether glycolytic reprogramming drives M1 polarization or is primarily a consequence of it—requires further experimental validation using gain- and loss-of-function approaches in relevant disease models. Both diseases cause macrophages to change their metabolism in response to stress; they shift into a state where they primarily use glucose for energy production for the purpose of producing pro-inflammatory signals and to facilitate damage to tissues, and both diseases have similar final common pathways of chronic inflammation and poor resolution. These processes sustain RA by maintaining synovitis, pannus development, cartilage breakdown, and bony erosions in the hypoxia of the synovial environment. Similarly, these processes lead to the inflammatory progression of atherosclerosis, leading to plaque instability and ultimately the acute coronary event that defines severe CHD. While the context of the two environments differs, the “immunometabolic logic” behind each is surprisingly consistent: increased inflammatory pressures, stress due to lack of nutrients, and alteration of signaling through pathways such as HIF-1α, AMPK, mTORC1, PI3K/AKT, and mitochondria progressively reduce the flexibility of macrophages and solidify harmful/ destructive phenotype(s).

The use of this framework also illustrates why patients with RA have an elevated cardiovascular burden that extends well beyond traditional CV risk factors. The chronic inflammatory process within the synovium (inflammation) does not remain localized to the joints; rather it generates a systemic inflammatory and metabolic response that can significantly enhance the development of atherosclerosis and increase the likelihood of developing coronary artery disease. In addition to being local effectors of tissue damage during inflammatory processes, macrophages may be cross-disease mediators that link the effects of both arthritic and vascular diseases through their involvement in similar patterns of metabolic dysfunction.

The focus on these metabolic pathways (i.e., those influencing M1/M2 macrophage polarization) lends traditional Chinese Medicine (TCM), and more broadly, pharmacognosy a potentially significant role in treatment. This is due primarily to the fact that several TCM compounds and formulae have demonstrated activity at sites along these pathways (e.g., ginsenoside Rb1; berberine) and/or with respect to related enzymes/pathways (e.g., HIF-1α, AMPK, mTORC1, PDK1). As such, TCM and other herbal-based treatments may be capable of providing more than simply anti-inflammatory benefit—they also may help restore a balance of metabolism, reduce an inflammatory state driven by high levels of glycolysis, and promote more reparative macrophage phenotypes within contexts of both CV disease and RA. While there is currently little robust mechanistic and clinical evidence supporting TCM use for these purposes, the biological plausibility of this approach has become increasingly supported by emerging data from the field of immunometabolism.
