# Correction: The dual role of metabolic reprogramming in macrophage polarization in rheumatoid arthritis and coronary heart disease and the intervention strategy of traditional Chinese medicine

**DOI:** 10.3389/fcvm.2026.1925661

**Published:** 2026-07-22

**Authors:** Haichun Zheng, Xiuyue Li, Jing Ru, Zhuoran Meng, Weibing Liu, Tao Jia, Shan Zhang, Qianwen Deng, Wenyi Huang, Renjie Dong, Qianrong Li

**Affiliations:** 1 School of Basic Medical Sciences, Yunnan University of Traditional Chinese Medicine, Kunming, Yunnan, China; 2 The First People’s Hospital of Yunnan Province, Kunming, China; 3 Department of Orthopedics, First Clinical Medical College of Yunnan University of Traditional Chinese Medicine, Kunming, Yunnan, China; 4 Yunnan Key Laboratory of Integrated Traditional Chinese and Western Medicine for Chronic Disease in Prevention and Treatment, School of Basic Medicine, Yunnan University of Chinese Medicine, Kunming, Yunnan, China

**Keywords:** atherosclerosis, coronary heart disease, macrophage polarization, metabolic reprogramming, rheumatoid arthritis, traditional Chinese medicine

Affiliation “Yunnan Key Laboratory of Integrated Traditional Chinese and Western Medicine for Chronic Disease in Prevention and Treatment, School of Basic Medicine, Yunnan University of Chinese Medicine, Kunming, Yunnan, China” was omitted from the paper. This affiliation has now been added for author Shan Zhang.

Affiliation “Department of Orthopedics, First Clinical Medical College of Yunnan University of Traditional Chinese Medicine, Kunming, Yunnan, China.” was omitted from the paper. This affiliation has now been added for author Tao Jia.

Author Tao Jia was erroneously assigned to affiliation “Yunnan University of Traditional Chinese Medicine, Kunming, China” This affiliation has now been removed for author Tao Jia.

Affiliation “School of Basic Medical Sciences, Yunnan University of Traditional Chinese Medicine, Kunming, Yunnan, China” was erroneously given as “Yunnan University of Traditional Chinese Medicine, Kunming, China”.

The original version of this article has been updated.

